# Quality indicators for colorectal cancer surgery and care according to patient-, tumor-, and hospital-related factors

**DOI:** 10.1186/1471-2407-12-297

**Published:** 2012-07-19

**Authors:** Simone Mathoulin-Pélissier, Yves Bécouarn, Geneviève Belleannée, Elodie Pinon, Anne Jaffré, Gaëlle Coureau, Dominique Auby, Jean-Louis Renaud-Salis, Eric Rullier

**Affiliations:** 1 Clinical and Epidemiological Research Unit, Institut Bergonié CRLCC, Bordeaux, France; 2 Inserm, CIC-EC7, Clinical Investigation Centre – Clinical Epidemiology, Bordeaux, France; 3 Université Bordeaux Segalen, 146 rue Léo Saignat, Bordeaux, France; 4 Department of Medical Oncology, Institut Bergonié, CRLCC, Bordeaux, France; 5 Department of Pathology, Hopital du Haut-Lévêque, CHU, Bordeaux, France; 6 Réseau de Cancérologie d’Aquitaine, Bordeaux, France; 7 Cancer register for Gironde, Bordeaux, France; 8 Department of Medicine, Centre Hospitalier Général, Libourne, France; 9 Department of Surgery, Hôpital Saint André, CHU, Bordeaux, France

**Keywords:** Cancer care, Cancer care organization, Colorectal cancer, Lymph node evaluation, Medical practice

## Abstract

**Background:**

Colorectal cancer (CRC) care has improved considerably, particularly since the implementation of a quality of care program centered on national evidence-based guidelines. Formal quality assessment is however still needed. The aim of this research was to identify factors associated with practice variation in CRC patient care.

**Methods:**

CRC patients identified from all cancer centers in South-West France were included. We investigated variations in practices (from diagnosis to surgery), and compliance with recommended guidelines for colon and rectal cancer. We identified factors associated with three colon cancer practice variations potentially linked to better survival: examination of ≥12 lymph nodes (LN), non-use and use of adjuvant chemotherapy for stage II and stage III patients, respectively.

**Results:**

We included 1,206 patients, 825 (68%) with colon and 381 (32%) with rectal cancer, from 53 hospitals. Compliance was high for resection, pathology report, LN examination, and chemotherapy use for stage III patients. In colon cancer, 26% of stage II patients received adjuvant chemotherapy and 71% of stage III patients. 84% of stage US T3T4 rectal cancer patients received pre-operative radiotherapy. In colon cancer, factors associated with examination of ≥12 LNs were: lower ECOG score, advanced stage and larger hospital volume; factors negatively associated were: left sided tumor location and one hospital district. Use of chemotherapy in stage II patients was associated with younger age, advanced stage, emergency setting and care structure (private and location); whereas under-use in stage III patients was associated with advanced age, presence of comorbidities and private hospitals.

**Conclusions:**

Although some changes in practices may have occurred since this observational study, these findings represent the most recent report on practices in CRC in this region, and offer a useful methodological approach for assessing quality of care. Guideline compliance was high, although some organizational factors such as hospital size or location influence practice variation. These factors should be the focus of any future guideline implementation.

## Background

The contrast between increasing colorectal cancer (CRC) incidence, which has increased by 50% in France between 1980-2000, and stable mortality can be attributed both to population ageing and to better survival, thanks to improved treatment for CRC [1]. Increasing numbers of patients are able to be resected for cure, diagnoses are made at earlier stages, operative mortality is decreasing and effective adjuvant treatments for patients stage III at diagnosis are now available. However, much variation exists in the way CRC is managed both internationally [2,3], and nationally within France [4-6], due partly to specific treatment modalities and their conformity to guidelines, and to structural factors such as accessibility, or to doctors’ willingness and competencies [7].

By reducing unwarranted variations in practice, guidelines should lead to better care, fewer inappropriate interventions and reduced costs [8]. However, active implementation is needed to increase adherence as publication and dissemination is not sufficient [9]. In France, as in other European countries, a national cancer plan has been implemented and adherence is promoted as an important step to enhancing quality of cancer care [10]. Regional cancer organizations (networks and local care units) have also been established to facilitate guideline use and to promote a multidisciplinary approach These procedural improvements have been put into practice in specialized centers progressively, but their impact has not yet been measured and no quality assessment data is available. Moreover, in the French health care system, CRC management is decentralized which can be expected to result in a multiplicity of treatment settings and specialist practices.

The aim of this article is to investigate and describe patterns of care management among a prospective cohort of primary CRC patients, just after implementation of regional clinical guidelines. We examine differences across patient-, tumor- and hospital-related factors using 27 criteria (11 for colon and 16 for rectal cancer) to describe the quality of colorectal cancer care. We also use statistical models to investigate factors linked to three specific quality indicators identified by the National Quality Forum/American College of Surgeons/Commission on Cancer and National Comprehensive Cancer Network (NCCN)/American Society of Clinical Oncology [11] and potentially linked to better survival: examination of ≥12 lymph nodes (LN) [12,13], use of adjuvant chemotherapy for stage II patients [14] and non-use for stage III patients [15].

## Methods

### Background

The Aquitaine region (South-West France) covers approximately 3 million inhabitants (5% of French population), including over 2,000 incident CRC cases. Based on national evidence-based guidelines for CRC management, a multidisciplinary group (38 physicians) implemented regional guidelines in 2003. Through regular meetings, and discussion, this group developed potential criteria to assess CRC care quality at four consecutive stages of patient care: diagnosis and preoperative work-up (10 criteria); surgery and pathological report (10 criteria); non-surgical treatments (4 criteria) and multidisciplinary team approach (3 criteria). (See Additional file 1: Appendix A). To disseminate the guidelines, we sent a global report (including recommendations and criteria) to the 10 local cancer units and the regional multidisciplinary team (150 practitioners). In addition, a guideline summary was sent to all registered regional physicians (about 6,000 general and specialist practitioners).

### Study population and data collection

All patients presenting at local cancer units with primary invasive colon or rectal cancer during the first year after guideline implementation (June 2003-June 2004) were included in this study. Patients were included prospectively and data were collected retrospectively. Cases were identified by surgeons, pathologists, or referring practitioners, or at multidisciplinary team meetings involving all 10 local cancer units.

Information was collected from patient medical files and pathology reports. This included demographic data (age, gender, place of residence), general status (ECOG score and comorbid conditions), clinical and surgical features of primary cancer (familial antecedents, localization, emergency or scheduled surgery, interventions, complications), diagnostic assessment (colonoscopy and biopsy, other exams), histological features of the tumor (histology, number of nodes examined, margin involvement, tumor stage), preoperative work-up (abdominal ultrasonography (US), thoracic chest X-ray, abdominal and thoracic computed tomography), surgical and adjuvant treatment (chemotherapy and radiotherapy), and multidisciplinary approach*.* Staging was based on the pathological report, and cancer extension was defined according to TNM classification as stage I (T1T2N0M0), stage II (T3T4N0M0) stage III (all T N1N2M0) and stage IV (all T, all N M1). The project was approved by the relevant French research committees (Commission Nationale Informatique et Liberté and the Comité Consultatif du Traitement Informatique en Recherche de Santé).

### Data and statistical analysis

In the first section, the quality of care is described using demographic data, tumor characteristics and 27 (11 rectal + 16 colon cancer) care management criteria (percentages). For patient age and management delays, we report medians. In the second section, statistical models are proposed for 3 specific survival-linked practices [12,13,15,16]: examination of ≥12 LNs, use of chemotherapy for stage II patients and non-use for stage III. Univariate analyses (χ2 or 2-sided Fisher's exact tests) were performed to identify associations between these three practice variation factors and the following factors: age (</≥75 years); location: main residence of patient (5 administrative districts), place of surgery (per administrative district) and hospital settings (public or private); performance status; comorbidities (antecedents and associated diseases); hospital volume for CRC procedures (</≥ 30 procedures per year); context of operative procedure (emergency/scheduled) and type of operative procedure (open, laparoscopy); tumor location; tumor stage and grade.

Multivariate logistic regression was then used to determine which variables were significantly and independently associated with ≥12 LN examination, chemotherapy use and non-use for stage II and stage III patients respectively. All statistically significant variables in the univariate analysis (p < 0.20) were entered simultaneously into the full model [17]. We also included potential confounding factors of age, stage and gender for the LN analyses and gender, age and grade for the two chemotherapy analyses, regardless of statistical significance. Using a manual backwards stepwise selection procedure, factors with higher p-values were removed until only variables with a p-value <0.05 were left in the final model [17]. Moreover, we tested modification effects adding interaction terms in the final model. None were statistically significant so they were not included in the final model. Results are expressed as odds ratios (OR) with their 95% confidence intervals (95%CI). Analyses were performed using SPSS Software (version 14.0).

Finally, we used data from the *Programme de Medicalisation des Systèmes d’Information (PMSI)* to compare our cohort to regional data according to setting, and district/area, according to numbers of patients and types of hospitals. Incidences from this regional administrative hospital and regional medical information system (further abbreviated as RMIS) have been validated against national incidences from the national register (FRANCIM) [18].

## Results

### Study population and settings

Overall, 1,411 patients were identified for inclusion across all centers. 205 patients were excluded (27 repeated inclusions, 30 misplaced medical records and 148 ineligible: 44 patients with surgery or multidisciplinary meeting outside of study inclusion; 34 T in situ (stage 0); 27 patients with relapse; 10 polyp or benign tumors, 6 lymphoma or neuro-endocrine tumors), leaving 1,206 pathologically-confirmed patients: 825 (68%) with a primary colon cancer and 381 (32%) with a primary rectal cancer (Table 1). Almost half (42%) of the cohort was ≥75 years. The majority of patients had one or more comorbid condition (68% colon, 66% rectal cancer). The most common comorbidity was cardiovascular disease (63% colon, 64% rectal cancer) but a large proportion of patients had an ECOG score 0-1 (66% and 80% for colon and rectal cancer respectively).

**Table 1:** Colon (n = 825) and rectal (n = 381) cancer patient characteristics in the study of surgery and care patterns for colorectal cancer (n = 1206)

**Characteristics**	**Colon**		**Rectal**	
	**N**	**(%)**	**N**	**(%)**
Median age	72 years	–	69 years	–
Gender				
Men	427	(52)	236	(62)
Women	398	(48)	145	(38)
Comorbid conditions (number)				
none	148	(18)	89	(23)
1	238	(29)	105	(28)
2	177	(21)	74	(19)
3 or more	147	(18)	74	(19)
Missing	115	(14)	39	(11)
ECOG Score				
0-1	541	(66)	303	(80)
>1	95	(12)	24	(6)
Missing	189	(23)	54	(14)
Family history of disease				
Yes	89	(11)	56	(15)
Missing	246	(30)	92	(24)
Colon tumor location				
Right	416	(50)	–	
Left	397	(48)	–	
Both	12	(2)	–	
Rectal tumor location				
Low	–		161	(42)
Median	–		129	(34)
High	–		91	(24)
Concomitant metastasis	216	(26)	83	(22)
Hospital setting at inclusion				
Public	414	(50)	187	(49)
Private	411	(50)	194	(51)
Teaching hospital	178	(22)	123	(32)
Non-teaching hospital	647	(78)	258	(68)
District of surgeon (or oncologist if no surgery)				
1	410	(50)	207	(54)
2	151	(18)	73	(19)
3	49	(6)	17	(5)
4	62	(7)	27	(7)
5	147	(18)	52	(14)
Other	6	(1)	5	(1)
Surgical procedures per year				
<30	97	(12)	25	(7)
30 and over	659	(80)	312	(82)
Outside area/no surgery	69	(8)	44	(12)

Patient care was distributed evenly across public and private hospitals (50% each), and around one quarter of patients came from teaching hospitals (1 comprehensive cancer center and 1 regional university hospital). Descriptive data on patients and hospitals from the RMIS over the same period indicate that our cohort was representative in terms of hospital district and setting (Additional file 1: Appendix B). Initial treatment was delivered in 53 hospitals or clinics with a median of 9.5 study patients per hospital over the one-year study period (interquartile range: 1.75-30, maximum 110).

### Patterns of care: diagnosis, surgery, pathology, adjuvant treatment and multidisciplinary approach

Considering only scheduled situations (683 colon and 368 rectal cancer patients), colonoscopy was performed for 91% of colon cancer patients and 87% of rectal cancer patients (biopsy for 85% and 96% respectively). Three hundred and twenty rectal cancer patients (87%) were locally staged either by a rectal US endoscopy, a scan or an MRI (following practices at the time). Specifically, 268 received a scan, 15 received MRI and 195 received transrectal US endoscopy, with some patients receiving more than one examination and a total 54% of patients receiving either an MRI or a transrectal US endoscopy. Concerning the pre-therapeutic work-up, an abdominal US or computed tomography was performed in 90% of colon cancer and 94% in rectal cancer patients.

Resection of the primary tumor (Table 2) was performed in 92% of colon cancer patients (of 825 patients overall, or 98% of the 767 patients receiving surgery) and 87% of rectal cancer patients (of 381 patients overall, or 97% of the 340 patients receiving surgery). Abdominal perineal resection was performed in 18% of rectal cancer patients. For patients without preoperative treatment, median delay between histological diagnosis and surgery was 17 days in colon cancer patients (532 patients) and 29 days in rectal cancer patients (125 patients).

**Table 2:** Surgical treatment and pathological report for colon (n = 767) and rectal (n = 340) cancer patients receiving surgery in the study of surgery and care patterns for colorectal cancer (n = 1107)

	**Colon**		**Rectal**	
	**N**	**(%)**	**N**	**(%)**
Mode of operation				
Emergency	134	(18)	12	(4)
Scheduled	617	(80)	325	(95)
Missing	16	(2)	3	(1)
Surgical access				
Open surgery	651	(85)	245	(72)
Laparoscopy	78	(10)	66	(19)
Transanal	0	–	12	(4)
Missing	38	(5)	17	(5)
Procedures				
Colectomy	755	(98)	–	–
Rectal resection	–	–	318	(94)
Colostomy alone	12	(2)	10	(3)
Transanal tumorectomy	–	–	12	(3)
Abdominoperineal resection				
Low	–	–	53	(16)
Mild/high	–	–	6/1	(2)
Post-operative 30-day mortality	32	(4)	8	(2)
Tumor grade				
Well or moderately differentiated	615	(80)	260	(76)
Poorly differentiated	89	(12)	26	(8)
Post-surgical stage				
I	82	(11)	85	(25)
II	261	(34)	75	(22)
III	233	(30)	99	(29)
IV	170	(22)	60	(18)
Missing	21	(3)	21	(6)
LNs (number examined)				
≥12	575	(76)	209	(66)
≥ 8	673	(89)	273	(86)
Median	17	–	14	–
Margin involvement (R1/(R0 + R1))*	10	(2)^¶^	38^†^	(19)

* n = 490 colon (colostomy alone, R2, RX and missing data excluded); n = 196 rectal (colostomy alone and transanal tumorectomy, R2 and RX excluded).

^¶^ : 480 patients R0; 10 R1 (19 R2; 2 Rx and 244 missing data excluded).

^†^ : 158 patients R0; 38 R1 (1 R2 and 119 Rx excluded).

Eight rectal cancer patients (unknown causes) and 32 colon cancer patients (3 anastomic fistulas, 1 abscess of the chest wall, 1 pulmonary embolism, 1 pro-lapse, 1 respiratory complication, 1 sub-occlusive syndrome and cystitis and 24 not specified) died within 30 days post-operatively. Postoperative mortality varied across surgery setting with 13% mortality (17 patients) in emergency settings and 2% in scheduled surgeries (15 patients, p < 0.001). Among surgical complications, clinical anastomotic leakage was the main complication, occurring in 5% of colon cancer patients (36 patients) and 10% of rectal cancer patients (26 patients).

Incomplete microscopic tumor resection was described for 2% (10 patients R1) of colon cancer patients. Circumferential margins for rectal cancer patients were provided in 65% of pathological reports (205 patients) and incomplete microscopic tumor resection was described in 19% of rectal specimens. At least 12 LNs were examined for 70.8% patients overall; 76% colorectal and 66% rectal.

Among colon cancer patients, adjuvant chemotherapy was performed in 26% of stage II (66/258 patients) and 71% of stage III (165/231 patients). For all patients, irrespective of stage of disease, chemotherapy with LV5FU2 (5-fluoruracil/ folinic acid) was widely used, alone or in association (stage II: 65/66 patients; stage III: 151/165 patients). Among rectal cancer patients, preoperative radiotherapy was performed in 84% of stage US T3T4 (177/212 patients), 87% of all N + patients (104/120) and 86% of all stage US T3T4 N + patients (101/117). Postoperative chemotherapy was administered in 67% of rectal cancer with node-positive US or pathology (92/138 patients).

Finally, a multidisciplinary team approach was taken before surgery for 14% of colon cancer patients (86/625 patients) and 54% of rectal cancer patients (175/327 patients). For rectal cancer patients, this approach was used in 67% when a pre-surgical treatment was performed (128/190 patients). After surgery, a multidisciplinary team approach was taken for 89% of colon cancer patients (685/767 patients) and for 80% of rectal cancer patients (273/340 patients). This results in over 85% of patients’ cases overall being discussed at some point in an multidisciplinary meeting.

### Variations in process quality measures in colon cancer patients

ECOG score, tumor localization, district for surgery and hospital volume were associated with examination of ≥ 12 LNs in univariate analyses. After adjustment (Table 3), the multivariate model showed that patients with ECOG score 1 (vs. score 0) (OR = 2.10; 95%CI 1.17-3.76), post-operative stage III (OR = 1.73; 95%CI 1.01-2.95) and higher hospital volume >30 procedures (OR = 3.34; 95%CI 1.70-6.56) were more likely to receive examination of ≥12 LNs. Left-sided colon cancer (OR = 0.58; 95%CI 0.34-0.97) and patients undergoing surgery in one of the five districts (OR = 0.38; 95%CI 0.17-0.85) were negatively associated with examination of ≥12 nodes.

**Table 3:** Factors associated with LN evaluation (LNE) or use of adjuvant chemotherapy (ACT) in colon cancer practices: uni- and multi-variate models

	**Univariate**			**Multivariate**		
	**OR**	**[95%CI]**	**p**	**OR**	**[95%CI]**	**p**
Evaluation of ≥ 12 LNs*	581 pts			399 pts		
ECOG score			0.011			0.014
0	1	ref		1	ref	
1	2.02	[1.20-3.41]		2.10	[1.17-3.76]	
2 and more	0.81	[0.41-1.60]		0.72	[0.33-1.55]	
Tumor location			0.003			0.036
Right	1	ref		1		
Left	0.54	[0.36-0.81]		0.58	[0.34-0.97]	
Post-operative stage			0.525			0.044
I -II	1	ref		1	ref	
III	1.14	[0.76-1.72]		1.73	[1.01-2.95]	
Hospital surgery volume			0.001			<0.001
< 30 operations	1	ref		1	ref	
≥ 30 operations	2.48	[1.46-4.21]		3.34	[1.70-6.56]	
Surgical district			<0.001			0.006
1	1	ref		1.00	ref	
2	1.62	[0.90-2.91]		2.07	[0.97-4.42]	
3	1.10	[0.48-2.51]		1.30	[0.35-4.88]	
4	0.34	[0.18-0.64]		0.38	[0.17-0.85]	
5	1.32	[0.73-2.40]		1.54	[0.84-3.40]	
Receiving ACT among stage II†	258 pts			255 pts		
Age (years)			<0.001			<0.0011
≥75	1	ref		1	ref	
< 75	8.68	[4.06-18.54]		28.49	[8.41-96.53]	
Local extension			<0.001			<0.001
pT3	1	ref		1	ref	
pT4	4.07	[2.12-7.81]		12.30	[3.81-39.75]	
Surgery context			<0.001			<0.001
Scheduled	1	ref		1	ref	
Emergency	6.89	[3.21-14.73]		25.31	[6.79-94.34]	
Hospital of surgery			0.009			0.020
Public	1	ref		1	ref	
Private	2.18	[1.21-3.95]		2.61	[1.16-5.87]	
Surgical district			0.090			0.036
1	1	ref		1	ref	
2	0.53	[0.25-1.13]		0.54	[0.19-1.53]	
3	1.89	[0.54-6.56]		1.45	[0.31-6.75]	
4	0.28	[0.06-1.29]		0.06	[0.08-0.39]	
5	0.57	[0.25-1.29]		0.44	[0.14-1.44]	
Not receiving ACT among stage III†	231 pts			226 pts		
Age (years)			<0.001			<0.001
< 75	1	ref		1	ref	
≥75	8.80	[4.51-17.17]		8.83	[4.34-17.95]	
Comorbid conditions			0.001			0.010
None	1	ref		1	ref	
One or more	3.46	[1.65-7.27]		3.04	[1.31-7.04]	
Hospitals			0.024			0.285
Private	1	ref		1	ref	
Public	1.94	[1.09-3.45]		1.48	[0.72-3.03]	

* Logistic model was adjusted by gender and age. Factors entered into the model but not retained are: department of patient residence, pre-operative colonoscopy, pre-operative abdominal ultrasound, usN, grade, type of colectomy. **†**ACT stage II and III: Logistic model was adjusted by gender and grade. Factors entered into the model but not retained for stage II patients: department of patient residence, comorbidities, family history of colorectal cancer; for stage III: family history of colorectal cancer and surgical district.

For stage II colon cancer patients, factors associated with chemotherapy use in the univariate analysis were younger age, advanced tumor stage, emergency context for surgery, and private hospital setting. In the multivariate analysis, the use of chemotherapy was associated with age <75 years (OR = 28.49; 95%CI 8.41-96.53), advanced tumor stage i.e. pT4 (OR = 12.30; 95%CI 3.81-39.75), emergency surgery (OR = 25.31; 95%CI 6.79-94.34) and private surgical hospitals (OR = 2.61; 95%CI 1.16-5.87). Adjuvant chemotherapy use was less likely in one of the five districts (OR = 0.06; 95%CI 0.08-0.39).

For stage III colon cancer patients, adjuvant chemotherapy was used for 89% of 134 patients < 75 years and for 47% of 97 patients ≥75 years (p < 0.001). Older age, presence of comorbidities and public hospitals were associated with the non-use (under-use) of adjuvant chemotherapy for stage III patients. The age effect and presence of comorbidities remained significant after multivariate analysis (OR = 8.83;95%CI 4.34-17.95; OR = 3.04, 95%CI 1.31-7.04 respectively).

## Discussion

This is the most recent study to report on patterns of colorectal cancer care in this French region. The majority of CRC patients were 70 years or older and most were treated in non-teaching hospitals. Overall, adherence to recommendations was good, but varied across hospitals and districts. For some important steps of cancer care (resection, pathology report, LN examination, chemotherapy in stage III, rectal radiotherapy), adherence was the same or higher than in other studies [19,20]. Over 70% of patients received standard care with chemotherapy for stage III colon cancer, although the proportion was lower for older patients. For rectal cancer patients, a majority (62%) of patients received preoperative radiotherapy which may reflect more frequent pre-operative use in Europe than in the United States where rates are reportedly on the rise [21]. Moreover, these results across an entire region in the year following guideline implementation show good adherence to the approach directly after. The main factors of non-compliance with recommendations were the high use of chemotherapy in stage II colon cancer and the under-use of diagnostic assessment for rectal cancer.

The examination of ≥12 LNs was associated with five factors: ECOG score, tumor characteristics (location and stage) and hospital characteristics (administrative district and volume of CRC procedures). These factors were concordant with almost all results published to date [12,22,23]. Pathological findings concerning resection specimens showed that for most CRC cases ≥12 LNs were examined, yet the proportion was slightly lower among rectal cancer patients compared to colon cancer patients. Radiotherapy before surgery may explain the lower proportion in rectal cancer patients due to reduced lymph node harvests post-radiotherapy [24,25]. Numerous studies and consensus guidelines have suggested that the 12 LN threshold is a reasonable minimum for adequate nodal evaluation in colon cancer, however there is still some uncertainty regarding the impact on survival [13,26,27].

To explain the under-use of chemotherapy in stage III, our study analyzed factors related to the potential risks of chemotherapy, finding very low use for patients over 75 years as has been reported in a similar population-based study [28]. While the efficacy of adjuvant therapy for stage III colon cancer was emphasized in the 1990s, age and comorbidity have always played a role in the decision to use adjuvant therapy in stage III [29], and it is mostly offered to healthy patients under 75 years old with recent results supporting the under-use for elderly patients [14,30].

Conversely, we chose to explain the determinants for using chemotherapy in stage II since at the time of study, adjuvant chemotherapy was generally not indicated in these cases. For stage II colon cancer, the present results show that in 2003-2004, younger age, advanced stage, emergency setting and healthcare organization (districts or sector hospitals) were associated with the use of chemotherapy. These results are in accordance with previous work showing that younger age, advanced stage, emergency presentation and discussion in multidisciplinary meetings were associated with adjuvant chemotherapy use for stage II patients [14]. At the time of this study, the national recommendations were not clear in terms of management options for stage II patients but these have since be clarified and adjuvant chemotherapy use appears to be indicated for specific stage II patients [31], although this still remains to be confirmed [32].

Although the volume-to-outcome relationship in CRC surgery seems smaller in magnitude than that in other digestive procedures, consideration must be given as to whether regionalization of CRC care is an appropriate mechanism for quality improvement [33]. In our study, we confirmed the importance of district as a source of heterogeneity in cancer colorectal management [4-6] This information could be a confounding factor with the hospital volume procedure, but we took this into account in our multivariate analysis and found an effect for both hospital district and volume. The hospital volume effect on examination of ≥12 LNs has been emphasized recently [23,34].

Our findings should be interpreted in the light of several limitations. First, our colorectal cohort was not exhaustive since the estimation of CRC incidence in this area was over 2000 new patients. However, similar patient and hospital characteristics were found in studies using data from French cancer registries or regional medical information systems [35,36]. A second limitation is our study size which is smaller than similar studies [22,23,37]. This lower power would explain why we did not identify stage as independent factors for LN examination. In addition, we had no information regarding patients’ socio-economic status [38] or treatment choice; two factors known to be relevant when explaining differences in care management. We also collected comorbidity information by using patients’ medical records, a method somewhat insufficient to capture the range and severity of the comorbidity.

In terms of interpreting whether the results reported here specifically reflect improvements after implementation of guidelines, with this observational design we cannot infer a definite causality link, although all efforts were undertaken to widely and comprehensively distribute guidelines as described in the methods. To provide an indication of trends in practices across time in France, we collated information from four comparable studies performed between 1900 and 2004 [4,14,36,39]. We note that the general trend for chemotherapy use in stage II patients did not change between 1990-1999 (21.8%) [4] to 2000 (20.4%) [14] and 2003/2004 (20.6%) in the present study. For stage III patients, the trend is towards an increase in the use of chemotherapy with usage reported at 47% in 1990-1999 [4], 61% in 2000 [14] and 71% in our study. Discussion in multidisciplinary meetings also appears to be on the rise. In 2000, between 32% [36] and 61% [14] of patients’ files were discussed in multidisciplinary meetings, compared to >85% in our series on the whole. Local population-based cancer registry data (unpublished data, http://www.registres-cancers-aquitaine.fr/) indicate that in one administrative district included in this region, discussion in multidisciplinary meetings for colorectal cancer patients increased from 48% to 63% between 2005 and 2008. We expect that with the creation of the National Institute for Cancer (INCa) in 2005 and standardized national guidelines, this trend is probably robust and continuous. Finally, trends in inspection of ≥12 LNs appear to be increasing with 45.2% compliance in 1997-2004 [40] compared to 70.8% in our study.

A final limitation is the differences in practices that may have occurred between time of observation and this publication, for example with magnetic resonance imaging practices. However, these data provide a validated measure of colorectal cancer care that may be applied in other regions and countries. The three NCCN quality indicators [11] examined here are supported as good measures of identifying practice variation that may be linked to survival differences.

## Conclusions

These findings show that there is a need to clarify underlying differences in practices observed not only on the basis of patients or tumor-related characteristics but also other factors, particularly hospital factors such as location and volume [41]. This is essential before comparing survival in CRC patients. Potential mechanisms underlying the relationships between volume and survival after colorectal surgery do not seem to have been characterized [42]. It is important to remember that many hospital-related factors, such as types of services, multidisciplinary teams and internal quality programs potentially explain differences in care management and cancer survival [43,44]. However, in 2009, the French cancer plan implemented a system authorizing cancer care only in hospitals above a specific volume threshold. For example, in digestive cancer surgery, the minimum number of cancer procedures is set at 30 per year [45], under which digestive cancer surgery is not authorized.

Overall, these data provide indication that adherence to clinical practice guidelines was relatively good in the year following their implementation by a multidisciplinary group. Since this date, the regional groups have established a quality of cancer care program for CRC in 2008 and new guidelines have been implemented, particularly for LN evaluation or the use of chemotherapy in certain stage II patients. A further study is underway using these updated criteria to further document practices in colorectal cancer care and report on survival (EvaCCoR).

In conclusion, this study provides good insight into real clinical practices (effectiveness) for a given population of patients treated in a designated geographical area when an experimental design is either not feasible or too complex to implement and hospital cohorts are not recommended. This point is particularly important for CRC because many patients are treated outside teaching hospitals.

## Abbreviations

CRC: Colorectal cancer; US: Ultrasonography; LN: Lymph nodes; OR: Odds ratio; 95%CI: 95% confidence interval; RMIS: Programme de Medicalisation des Systèmes d’Information (PMSI); FRANCIM: France-cancer-incidence et mortalité; ECOG: Eastern Cooperative Oncology Group; NCCN: National Comprehensive Cancer Network.

## Competing interests

The authors declare that they have no competing interests.

## Authors’ contributions

SMP participated in the conception, design, acquisition of data and analysis and interpretation, and in the drafting and approval of the final manuscript. YB participated in the conception and design of the study and in the drafting and approval of the final manuscript. GB participated in the acquisition of data and analysis and interpretation, and the drafting and approval of the final manuscript. EP - acquisition of data and analysis and interpretation, and the drafting and approval of the final manuscript. GC - acquisition of data and analysis and interpretation, and the drafting and approval of the final manuscript. AJ participated in the conception, design of the study and the drafting and approval of the final manuscript. DA participated in the conception, design of the study and the drafting and approval of the final manuscript. JLRS participated in the conception, design of the study and the drafting and approval of the final manuscript. ER participated in the conception, design of the study and the drafting and approval of the final manuscript.

## Acknowledgements

The authors thank I. Cirilo-Cassaigne for participation in data collection, physicians of the cancer units, the Aquitaine Gastro Association, the Committee of Regional Medical Information for regional data, the “Ligue contre le cancer” for financial support and Pippa McKelvie-Sebileau, employee of Institut Bergonié, for medical writing assistance.

## GRACCOR

S Abdiche; X Adhoute; JC Arnal; R Arotçarena; D Auby; F Audemar; A Avril; C Balabaud; C Baldit; J Bancons; E Barandon; C Barberis; J Bayle; B Bayol; R Belliard; P Berthélémy; L Berthoux; R Beyssac; JF Blanc; C Boisseau; N Bonichon; P Bonichon; F Boudinet; C Bouet; P Boutillier; M Breque; F Bretagnol; G Breuillé; H Brocard; E Brudieux; R Brunet; E Buy; J Calabet; V Calès; L Cany; J Carles; B Carles; R Cayla; JB Cazals; JL Cazenave; JL Cazenave; JP Cazenave-Mahe; S Cazorla; JB Chacon; P Champbenoit; P Chastan; C Chaussende; I Chossat; M Claracq; D Collet; D Coomans; JL Coquard; F Cordet; B Couderc; P Couzigou; O Dahan; B Dantin; J Dauba; XR David; A deMascarel; B Debenes; D Delvert; C Deret; D Desprez; N Dohollou; C Dost; C Dromer; V Dubuisson; F Dumas; V Dumora; E Echinard; D El Kohen; S Evrard; O Fitoussi; M Fonck; C Fromenteau; T Gaultier; L Gauriau; B Gheysens; B Goffre; R Gontier; JB Griot; JP Guichandut; F Guichard; A Humbert; Y Imbert; D Jaubert; F Junes; B Kin; J Labat; Y Laborde; A Lamy; D Larregain-Fournier; D Larroude; PH Larrue; C Laurent; G Le Roux; N Le Toux; L Le Trong; R Lecesne; P Ledaguenel; A Lepoutre; Letout; CB Levache; AM Lévêque; P Lotte; I Lariviere; S Loze; R Lupo; K Machané; E Magne; F Magnien; P Mahé; N Mallier; PR Mannant; JL Manouvrier; F Marty; B Masson; O Maton; JC Mauriac; F Minet; F Miremont; D Moussié; F Ndobo; S Nobili; D Noury; J Ogouchi; B Oui; M Pansieri; Y Parent; A Pariente; P Peluchon; JF Pichon; B Prevost; P Puech; J Pujol; H Rallier; A Rault; G Reboul; P Remuzon; S Rémy; B Richard-Molard; P Roussy; D Roux ; A SaCunha; P Santoni; J Saric; M Sarkissian; JC Schang; G Simon; D Smith; P Stépani; P Talbi; P Texereau; N Trufflandier; K Turner; V Vendrely; JF Vergier; F Weber

Supported by a 2001 PHRC grant (Programme Hospitalier de Recherche Clinique) from the French Health Ministry.

## Pre-publication history

The pre-publication history for this paper can be accessed here:

http://www.biomedcentral.com/1471-2407/12/297/prepub

## Supplementary Material

Additional file 1 **Appendix A. Criteria to assess the quality of colorectal cancer care at four consecutive management steps as defined by the multidisciplinary group for Colorectal cancer (38 experts).** Appendix B. Comparison between district area, hospital setting and surgical procedure volume (colon and rectal surgery) in the regional medical information system (RMIS) and our regional cohort (June 2003-June 2004), Aquitaine area. Click here for file
