# Supplementary material for: Quality indicators for colorectal cancer surgery and care according to patient-, tumor-, and hospital-related factors
Source: BMC Cancer. 2012 Jul 19;12:297. doi: 10.1186/1471-2407-12-297 (PMC3527146; doi:10.1186/1471-2407-12-297)
Supplement: Additional file 1 — Appendix A. Criteria to assess the quality of colorectal cancer care at four consecutive management steps as defined by the multidisciplinary group for Colorectal cancer (38 experts). Appendix B. Comparison between district area, hospital setting and surgical procedure volume (colon and rectal surgery) in the regional medical information system (RMIS) and our regional cohort (June 2003-June 2004), Aquitaine area. [file 1471-2407-12-297-S1.doc]

Appendix A. Criteria to assess the quality of colorectal cancer care at four consecutive management steps as defined by the multidisciplinary group for Colorectal cancer (38 experts)

| **Colon cancer** | **Rectal cancer** |
| --- | --- |
| **Diagnosis and preoperative work-up** | |
| 1. Clinically diagnosed cancers with biopsies from endoscopy | 1. Clinically diagnosed cancers with biopsies from endoscopy |
|  | 2. Patients receiving a rigid rectoscopy during the pre-treatment work-up |
| 2. Patients receiving a complete colonoscopy before the surgical treatment | 3. Patients receiving a complete colonoscopy before the surgical treatment |
|  | 4. Patients receiving an ultrasound-guided rectal endoscopy during the pre-treatment workup |
| 3. Patients receiving an abdominal ultra-sound during the pre-treatment workup | 5. Patients receiving an abdominal ultra-sound during the pre-treatment workup |
| 4. Patients receiving a pulmonary radiography during the pre-treatment work-up | 6. Patients receiving a pulmonary radiography during the pre-treatment work-up |
| **Surgery and pathological report** | |
|  | 7. Abdominal-perineal amputations with repartition by thirds (high, intermediate and lower rectum) |
|  | 8. Quality of the surgery |
| 5. Symptomatic anastomotic fistula | 9. Symptomatic anastomotic fistula |
| 6. Post-operative mortality (within 30 days) | 10. Post-operative mortality (within 30 days) |
| 7. Patients for whom 12 or more LNs are examined | 11. Patients for whom 12 or more LNs are examined |
| 8. Complete pathological reports | 12. Complete pathological reports |
| **Multidisciplinary team approach** | |
|  | 13. Patient’s medical file discussed in multidisciplinary meeting before surgery |
| 9. Patient’s medical file discussed in multidisciplinary meeting after surgery | 14. Patient’s medical file discussed in multidisciplinary meeting after surgery |
| **Non-surgical treatments** | |
| 10. Patients with stage II cancer not receiving post-operative chemotherapy |  |
| 11. Patients with stage III cancer receiving post-operative chemotherapy |  |
|  | 15. Patients with a T3 or T4 tumor receiving pre-operative radiotherapy |
|  | 16. Patients with a usN+ or pN+ tumor receiving post-operative chemotherapy |

Appendix B. Comparison between district area, hospital setting and surgical procedure volume (colon and rectal surgery) in the regional medical information system (RMIS) and our regional cohort (June 2003-June 2004), Aquitaine area

|  | Hospitals | | | |  | Patients | | | |
| --- | --- | --- | --- | --- | --- | --- | --- | --- | --- |
|  | RMIS (93) | | Cohort (43)* | |  | RMIS (2,239) | | Cohort (1,064)* | |
|  | N | (%) | N | (%) |  | N | (%) | N | (%) |
| District |  |  |  |  |  |  |  |  |  |
| 1 | 36 | (39) | 19 | (44) |  | 1064 | (48) | 558 | (52) |
| 2 | 13 | (14) | 5 | (12) |  | 243 | (11) | 195 | (18) |
| 3 | 9 | (10) | 5 | (12) |  | 191 | (9) | 59 | (6) |
| 4 | 13 | (14) | 4 | (9) |  | 206 | (9) | 77 | (7) |
| 5 | 22 | (24) | 10 | (23) |  | 535 | (24) | 175 | (16) |
| Hospital setting |  |  |  |  |  |  |  |  |  |
| Private | 50 | (54) | 25 | (58) |  | 1135 | (51) | 567 | (53) |
| Public | 43 | (46) | 18 | (42) |  | 1104 | (49) | 497 | (47) |

** 1,085 patients with surgery (755 colon and 330 rectal cancers) but 21 patients with missing data or hospitals outside the area (10 hospitals)*
